# Neuroendocrine (Merkel cell) carcinoma of the retroperitoneum with no identifiable primary site

**DOI:** 10.1186/1477-7819-5-117

**Published:** 2007-10-19

**Authors:** Van Boghossian, Ira D Owen, Balakrishna Nuli, Philip Q Xiao

**Affiliations:** 1 Department of Surgery, Brooklyn Hospital Center, 121 Dekalb Ave., Brooklyn, New York, 11201 USA; 2 Department of Pathology, Brooklyn Hospital Center, 121 Dekalb Ave., Brooklyn, New York, 11201 USA

## Abstract

**Background:**

Neuroendocrine carcinoma is an aggressive neoplasm that mainly affects elderly Caucasians and typically arises in sun-exposed areas of the skin. The disease is rather rare and only a relatively few cases present with no apparent primary lesion.

**Case presentation:**

We report a case of an 81-year-old Caucasian male with neuroendocrine carcinoma, which initially presented as a large retroperitoneal mass. Pathological and immunohistochemical analysis of the transabdominal CT-guided biopsy specimen revealed tissue consistent with neuroendocrine carcinoma. The patient underwent exploratory laparotomy and the mass was successfully excised along with an associated mesenteric lymph node.

**Discussion:**

There are currently two possible explanations for what occurred in our patient. First, the retroperitoneal mass could be a massively enlarged lymph node where precursor cells became neoplastic. This would be consistent with a presumptive diagnosis of primary nodal disease. Alternatively, an initial skin lesion could have spontaneously regressed and the retroperitoneal mass represents a single site of metastasis. Since Merkel cell precursors have never been identified within lymph nodes, the latter theory seems more befitting. Moreover, metastasis to the retroperitoneal lymph nodes has been reported as relatively common when compared to other sites such as liver, bone, brain and skin.

**Conclusion:**

Wide local excision of the primary tumor is the surgical treatment of choice for localized disease. We propose that further studies are needed to elucidate the true efficacy of chemotherapy in conventional as well as unconventional patients with neuroendocrine carcinoma.

## Background

Neuroendocrine carcinoma, also known as Merkel cell carcinoma, is an aggressive neoplasm that mainly affects elderly Caucasians and typically arises in sun-exposed areas of the skin. In this report, we examine a rare case of neuroendocrine carcinoma, which initially presented as a retroperitoneal mass. After extensive workup, no primary site was identified in the skin or elsewhere. Relatively few cases present with no apparent primary lesion.

## Case presentation

An eighty-one year old Caucasian male presented in June 2006 to the Emergency Department of the Brooklyn Hospital Center with a one-month history of blood- tinged stools. He was admitted to the medical service for further management. The surgical team was consulted for evaluation of pain associated with a palpable mass in the right iliac fossa. The pain was described as dull, non-radiating and constant in nature. It was rated a 3 on a 10-point intensity scale and there were no reported exacerbating or ameliorating factors. The patient's history was significant for intermittent constipation and repair of an incarcerated umbilical hernia three weeks prior.

**Figure 1:**
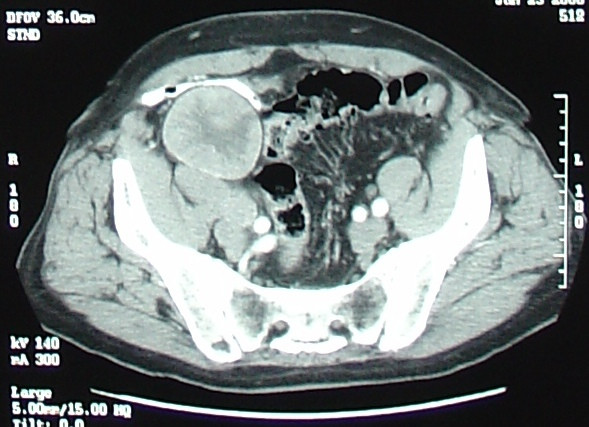
CT scan of pelvis showing right lower quadrant mass.

On examination, vital signs were normal. The abdomen was non-distended with a 3-cm, midline, infraumbilical scar. Tenderness was noted on deep palpation of the right lower quadrant and a large mass was felt measuring approximately 5-cm × 5-cm. The lower margin could not be felt. The mass was also palpable on digital rectal exam. Femoral pulses were normal. Laboratory values were unremarkable. Fecal occult blood test was negative. Colonoscopy showed the cecum high in the right upper quadrant with no evidence of a cecal mass. Contrast computed tomography (CT) of both abdomen and pelvis (Figure 1) revealed a 5-cm × 5-cm × 7.5-cm, enhancing, rounded, heterogeneous, well-defined mass in the right lower quadrant, ventral to the psoas muscle and displacing the terminal ileum anteriorly.

**Figure 2:**
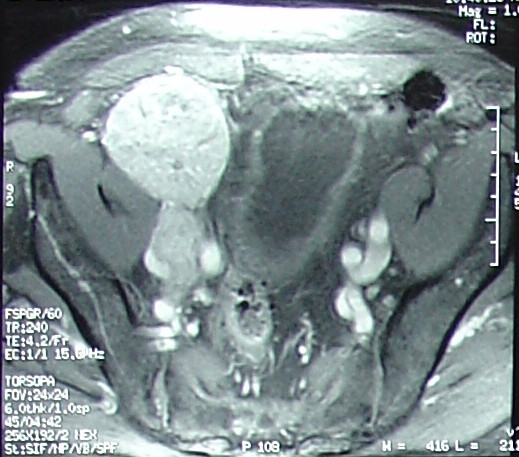
MRI of pelvis showing right lower quadrant mass compressing external iliac vessels.

**Figure 3:**
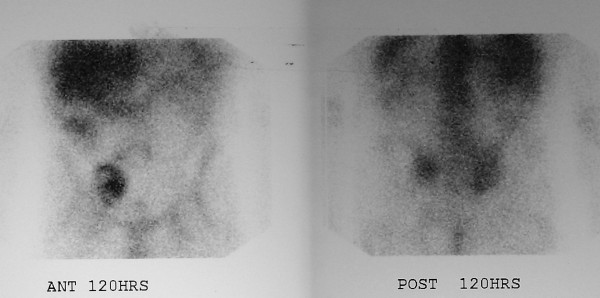
Gallium scan showing right lower quadrant enhancement.

**Figure 4:**
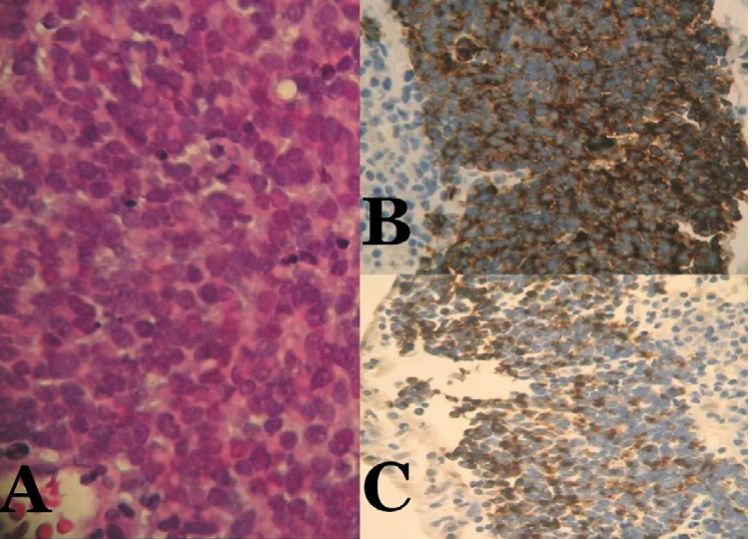
Microscopic examination reveals tumor composed of monotonous round cells showing scant eosinophilic cytoplasmic rim, round and vesicular nuclei with finely granular and dusty chromatin and multiple nucleoli (A, hematoxylin and eosin ×100). Tumor cells are positive for synaptophysin (B, ×100) and CK 20 (C, ×100).

Chest X-ray was unremarkable. Magnetic resonance imaging (MRI) of the pelvis (Figure 2) confirmed CT findings and showed that the mass abutted and compressed the external iliac vessels. Pseudoaneurysm was ruled out. Gallium and positron emission tomography (PET) scans (Figure 3) revealed localized abnormal activity in the right lower quadrant. Octreoscan showed a solitary, abnormal lesion in the lower right quadrant consistent with previous findings. No other lesions, primary or metastatic, were demonstrated in any of these studies. Meticulous physical examination of the skin and lymph nodes also revealed no suspicious lesions. There was no evidence of appendicitis and biopsy was recommended. Exhaustive pathological and immunohistochemical analysis of the transabdominal CT-guided biopsy specimen (Figure 4) revealed that tumor cells were positive for both synaptophysin and CK 20, but negative for TTF-1, CK7, WT-1, HBME-1, CD45, CD20, CD3, PAP, PSA, S100 and HMB-45. With such a staining profile, a definitive diagnosis of neuroendocrine (Merkel cell) carcinoma was made and we chose to forego further analysis.

The patient then underwent exploratory laparotomy and the mass was successfully excised along with an associated mesenteric lymph node. Final pathologic analysis of the mass was consistent with biopsy findings and the mesenteric lymph node was negative for tumor. Extubation was delayed due to difficult intubation and the patient was transferred to the surgical intensive care unit for observation. He was extubated the following day and transferred to the surgical floor where he had an uneventful recovery and was discharged on postoperative day eight. The patient's care was then transferred to an oncologist at another institution where he is currently being monitored and evaluated for treatment with various chemotherapeutic regimens.

## Discussion

Neuroendocrine carcinoma is an aggressive, usually cutaneous neoplasm with a propensity for metastasis. The tumor mainly affects elderly Caucasians. Although it is widely referred to as Merkel cell carcinoma, the term neuroendocrine carcinoma of the skin is more appropriate due to the tumor's histologic and histochemical characteristics [1]. The disease is rather rare with an annual incidence ranging from 0.2 to 0.45 cases per 100,000 [2-4]. In a review of 661 cases conducted in 2000, only 2 percent presented with no apparent primary lesion [5]. However, in a later, but smaller series, 19% of those with nodal neuroendocrine tumors had no detectable primary site [6].

Merkel cells reside in the basal layer and hair follicles of the skin's epidermis and are associated with mechanoreceptors in the dermal papillae [7]. It has been proposed that neuroendocrine carcinoma of the skin arises from these cells. However, since it is mainly a dermal tumor, an alternative hypothesis that they originate from immature totipotent stem cells and acquire neuroendocrine characteristics upon malignant transformation [1,8,9] is perhaps more plausible. The occasional presence of squamous or eccrine differentiation in these tumors also suggests stem-cell origin [10].

Sun exposure is thought to play a major role in pathogenesis, but some cases present with lesions in non-sun-exposed areas. In our case, sun-exposure seems to have played no role, as the tumor apparently did not even involve the skin. Immunosuppression can also predispose to the disease, but it was not a factor in this case since the patient was not immunosuppressed. There are currently two possible explanations [1,11-13] for what occurred in our patient. First, the retroperitoneal mass could be a massively enlarged lymph node where precursor cells became neoplastic. This would be consistent with a presumptive diagnosis of primary nodal disease. Alternatively, an initial skin lesion could have spontaneously regressed and the retroperitoneal mass represents a single site of metastasis. Since Merkel cell precursors have never been identified within lymph nodes, the latter theory seems more befitting. Moreover, metastasis to the retroperitoneal lymph nodes has been reported as relatively common when compared to other sites such as liver, bone, brain and skin [9].

It is difficult to accurately diagnose neuroendocrine carcinoma due to its similarity to other poorly differentiated "small blue cell tumors" like small cell carcinoma of the lung [14]. Histopathologic differentiation techniques are necessary for definitive diagnosis. When found, perinuclear keratin filaments on electron microscopy and a dot-like pattern with cytokeratin (CK) 20 and CK 7 staining aid in diagnosis [15-17].

Staging is done according to criteria developed by the Memorial Sloan-Kettering Cancer Center [18]. Stage I describes node-negative disease with a tumor less than 2-cm in size. Node-negative disease with tumors equal to or greater than 2-cm in size is classified as Stage II. Stage III represents nodal metastasis and stage IV represents distant metastasis. Workup for staging includes chest radiograph to rule out small cell lung cancer and CT scan of the chest and abdomen.

In 2005, Allen et al reported five-year disease-specific survival for patients with neuroendocrine carcinoma to be 64% [18]. In 2006, Clark et al found disease-specific survival at five years to be 49% and overall survival at five years to be 62% [11]. In most cases, these tumors are aggressive and have a high rate of metastasis and recurrence. Survival rates for patients with disease beyond the primary lesion are comparable to those of patients with malignant melanoma, nodal spread being the best predictor of distant metastatis or death [18]. Primary nodal neuroendocrine carcinoma follows a less aggressive course than the metastatic form of nodal involvement [19]. Thus, follow-up of patients may provide insight into whether nodal disease was primary or metastatic.

The aggressive nature of this disease necessitates frequent follow-up. The presence of risk factors including tumors larger than 2-cm, truncal location, male sex, age over 65, nodal or distant disease at presentation and duration of disease before presentation [5] should determine the appropriate frequency. On examination, the clinician should focus on the lymphatic and integumentary systems. When symptoms lead to suspicion of recurrence, appropriate imaging studies should be performed.

## Conclusion

Wide local excision of the primary tumor is the surgical treatment of choice for localized disease. This approach along with nodal dissection and locoregional irradiation in clinically node-positive patients or elective lymph node resection in clinically node-negative patients has been associated with both decreased rate of and longer interval to recurrence. The value of chemotherapy is still undetermined [20]. Thus, our patient received none. Since our patient's tumor could have originated from a lymph node, six weeks of loco-regional irradiation was administered following wide local excision. We propose that further studies are needed to elucidate the true efficacy of chemotherapy in patients with conventional as well as unconventional presentations of neuroendocrine carcinoma.

## Competing interests

The author(s) declare that they have no competing interests.

## Authors' contributions

VB interpreted data and critically revised the manuscript. IDO reviewed the literature, prepared the manuscript and obtained patient consent. BN provided the case and critically revised the manuscript. PQX provided the pathological images and assisted in interpreting them. All authors read and approved the final manuscript.
